# Modelling Blow Fly (Diptera: Calliphoridae) Spatiotemporal Species Richness and Total Abundance Across Land-Use Types

**DOI:** 10.3390/insects15100822

**Published:** 2024-10-20

**Authors:** Madison A. Laprise, Alice Grgicak-Mannion, Sherah L. VanLaerhoven

**Affiliations:** 1 Department of Integrative Biology, University of Windsor, Windsor, ON N9B 3P4, Canada; madison.laprise@gmail.com; 2 School of the Environment, University of Windsor, Windsor, ON N9B 3P4, Canada

**Keywords:** Diptera: Calliphoridae, multivariate regression models, geospatial technology, multidisciplinary, entomology, sampling, species distributions

## Abstract

Geographic Information Systems provide the means to explore the spatial distribution of insect species across various land-use types to understand their relationship with shared or overlapping spatiotemporal resources. Blow fly species richness and total fly abundance were correlated among six land-use types (residential, commercial, waste, woods, roads, and agricultural crop types) and distance to streams. To generate multivariate models of species richness and total fly abundance, blow fly trapping sites were chosen across the land-use gradient of Windsor–Essex County (Ontario, Canada) using a stratified random sampling approach. Sampling occurred in mid-June (spring), late August (summer), and late October (fall). Spring species richness correlated highest to residential (−), woods (−), distance to streams (+), and tomato fields (+) in models across all three land-use buffer scale distances (0.5, 1, 2 km), with waste (+/−), roads (−), wheat/corn (−), and commercial (−) correlating at only two of the three scales. Spring total fly abundance correlated with all but one land-use variable across all buffer scale distances, but the distance to streams (+), followed by orchards/vineyards (+) exhibited the greatest importance to these models. Summer blow fly species richness correlated with roads (−) and commercial (+) across all buffer distances, whereas at two of three buffer distances wheat/corn (−), residential (+), distance to streams (+), waste (−), and orchards/vineyards (+) were also important. Summer total fly abundance correlated to models with distance to streams (+), orchards/vineyards (+), and sugar beets/other vegetables (+) at the 2 km scale. Species richness and total abundance models at the 0.5 km buffer distance exhibited the highest correlation, lowest root mean square error, and similar prediction error to those derived at larger buffer distances. This study provides baseline methods and models for future validation and expansion of species-specific knowledge regarding adult blow fly relationships with spatiotemporal resources across land-use types and landscape features.

## Simple Summary

Geographic Information Systems provide the means to examine the distribution of insect species that recycle nutrients in our environment. Blow flies were trapped using liver-baited bottle traps across Windsor–Essex County (Ontario, Canada), with sites randomly chosen using geospatial mapping technology, and compared to land-use types. In the spring (mid-June), the number of blow fly species was lower near residential, commercial, wooded areas, roads, and wheat/corn fields, but higher near streams and tomato fields. Waste areas both increased and decreased the number of blow fly species, depending on whether land-use categories were considered at distances of 0.5, 1, or 2 km from the trapping site. In the summer (late August), the number of blow fly species was lower near roads, waste, and wheat/corn fields but higher near commercial, residential, orchards/vineyards, and streams. In both spring and summer, the highest overall number of blow flies were trapped near streams, orchards/vineyards, and specifically in summer, near sugar beets/other vegetable fields. This study provides baseline methods to examine blow fly use of different foods across time and space as influenced by humans, and initial insights into how our choices to modify the landscape impact the distribution of these important insects.

## 1. Introduction

Blow flies (Diptera: Calliphoridae) are pests of livestock and stored meats [1], carriers of disease [2] and important decomposers for recycling nutrients [3]. Blow fly species that consume carrion in their larval stages can be used as evidence in forensic investigations [4]. They are often observed when studying the death and decay of animal matter and have been model specimens when studying population dynamics [5]. Within Canada, there are 62 Calliphoridae species at present [6], with 20 carrion-consuming species currently known from across Eastern Canada, ranging from the Arctic to the border with the United States [7]. In a 2005 study of Calliphoridae species diversity within Windsor–Essex County (Ontario, Canada) across spring, summer, and fall, 11 of these carrion-consuming species were collected from forested and open-field sites [8].

The coexistence theory [9] suggests that in patchy, ephemeral resource-based systems such as those based on carrion, species potentially have a high risk of extinction based on their non-equilibrium patch dynamics and unpredictability of resources in natural environments. Yet, habitat fragmentation studies have revealed that the anthropogenic modification of landscapes changes the distribution of resources and can modify the resource itself. In the case of carrion utilized by blow flies as oviposition sites for offspring development and protein for adult maturation of eggs and sperm, we propose that human habitation creates reliable patches of decomposing remains through garbage bins, waste-processing sites, landfills, animal livestock production facilities and roadkilled animals associated with vehicular traffic. Thus, land use may be a useful proxy of carrion availability for assessing and predicting blow fly diversity. Furthermore, land use may provide insight into other sources of protein for gonad maturation and sugars for energy that are critical for adult blow flies [10].

Few studies have measured the spatiotemporal distribution of blow flies with respect to detailed land use or landscape characteristics globally. Often, these studies only assess blow fly distribution using more simplified categories of shaded or sunny [11,12], urban or rural [13,14], urban, periurban, and forest [15], or urban, rural, and mixed [16], and seasonal preference [17]. One study conducted in Northern Thailand examined the distribution of four blow fly species across six habitat types of disturbed deciduous forest, mixed deciduous forest, mixed orchard, paddy field, lowland village, and city town, with some preferences demonstrated for mixed deciduous forest [18]. A study conducted using urban and rural sites across Canada collected 14 species at rural sites, and only eight of those species at urban sites [13], with the conclusion that those blow fly species that prefer urban locations may have adapted as urban specialists [13]. Yet, an urban habitat type could correspond to various land-use/landscape elements such as residential areas, commercial areas, highways, waste facilities, parks with forested areas or riversides. It begs the question of what diversity of land-use/landscape elements comprise the necessary urban area to no longer support blow fly species found in surrounding rural areas.

The association of blow flies with some specific land-use elements has been demonstrated or is the logical conclusion of other studies. It is no surprise that waste facilities such as landfills have the potential to be breeding grounds for a variety of flies and other insects. One study that looked at nuisance flies found that within landfills the most abundant flies were *Musca domestica* L. (Diptera: Muscidae), while blow flies were in lower numbers, but as distance from the landfill increased into the surrounding residential areas, the number of blow flies identified doubled [19]. Blow flies are important pollinators for some vegetable and fruit crops [20,21,22,23] and have been found to be as effective as bees in some instances [23]. Thus, agricultural fields may provide beneficial resources for blow flies, supporting their diversity. Yet, the specific crop types are likely to influence this relationship as not all crops provide pollen and/or nectar accessible to these flies. Roadkill is a direct source of carrion for blow flies. Roadkill prevention studies, such as that by Lin (2016) [24], have found that reduced traffic volumes and lower speed limits were the most effective methods of decreasing roadkill; thus, high-traffic, higher speed limit roads, such as highways and expressways, should have higher rates of roadkill available and a higher diversity of blow flies.

We used a stratified random sampling strategy of baited traps across specific land-use and landscape parameters including residential, commercial (including restaurants and other businesses), wooded areas, agricultural crops, landfill and waste treatment areas, three levels of traffic and road speed, and reported roadkill to measure blow fly diversity during the spring, summer, and fall. Using three buffer diameter sizes of 0.5, 1, and 2 km around each site, we developed a geodatabase of area and distance parameters within these buffers that, in addition to the above variables, also included distance to the nearest stream, total road length, and agricultural crop types divided into (1) orchards/vineyards; (2) wheat/corn/oats; (3) soybean/dry bean/canola; (4) tomatoes/potatoes; and (5) sugar beets/other vegetables. Our objective was to develop multivariate landscape regression models of the relationship between land-use/landscape parameters as potential predictors of blow fly spatiotemporal species richness and total fly abundance.

## 2. Materials and Methods

### 2.1. Sampling Location

Windsor–Essex County, Ontario, is located at the southernmost point of Canada, covering approximately 1851 km^2^ of land. Its eastern border is adjacent to the Detroit, Michigan, USA border, running along the Detroit River. Windsor–Essex County’s northern border is surrounded by Lake Saint Clair and its southern border by Lake Erie. This, coupled with warm summer temperatures, creates high humidity conditions. The Windsor–Essex area experiences relatively mild winters for Canada, typically reaching an average temperature of −4 °C and average relative humidity of 77%, and warm humid summers reaching approximately 23 °C, with average relative humidity levels of 86%. Geographically this region is composed predominantly of agricultural land surrounding small towns, Essex County has approximately 1328 km^2^ of agricultural land [25]. Windsor–Essex County encompasses eight different municipalities: Amherstburg, Essex, Kingsville, Lakeshore, LaSalle, Leamington, Tecumseh, and Windsor. Windsor is the largest city among the municipalities within Essex County, with a population of approximately 217,188 [26]. In total, Essex County (including the City of Windsor) has an approximate population of 329,144 [26].

### 2.2. Data Source Criteria

The following criteria were chosen and included in a geodatabase to examine perceived adult blow fly habitat prevalence due to their assumed relationship production or availability of carrion, protein sources (meat, feces, and pollen), and/or sugar sources (fruit, nectar, aphid honeydew, and anthropogenic-derived sugars). In addition to traffic volume and reported roadkill, six land-use types were chosen to develop the sampling strategy for blow flies: (1) Residential (areas where people reside); (2) Commercial (areas consisting primarily of businesses and restaurants); (3) Wooded Areas (areas with any type of tree cover or vegetation that exceeds a height of 2 m); (4) Livestock Farms (horse and cattle farms); (5) Agricultural Crops, and (6) Waste (Landfills and waste treatment centers).

### 2.3. Sampling Strategy and Spatial Criteria

**Figure 1:**
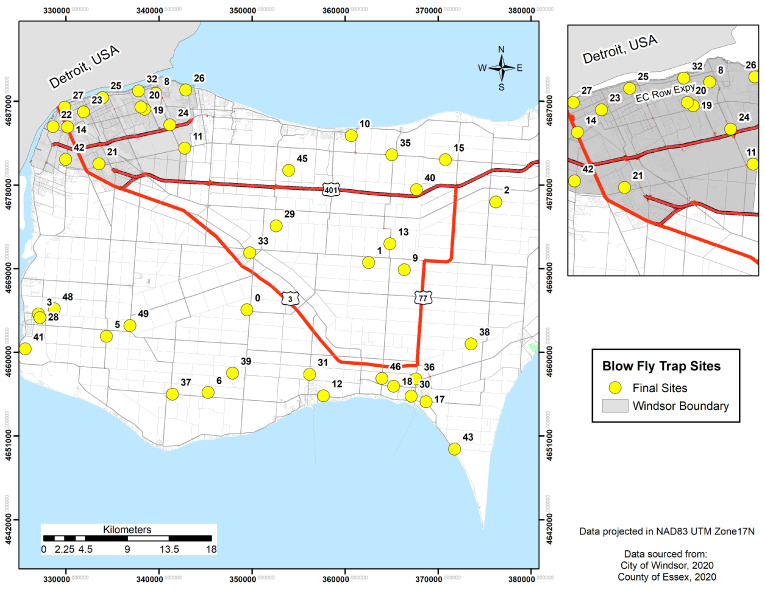
Blow fly sampling locations in Windsor–Essex County, Ontario, Canada spread across land-use types of residential, commercial, wooded, waste, and livestock farms, three levels of road speed, and three levels of reported roadkill densities.

A total of 50 locations were selected using a stratified random sampling approach developed using land-use type geodatabases, traffic volume levels, and dead animal removal levels (Figure 1). Three of these fifty sites were deemed filler points, and their purpose was to fill in any gaps on the map once the sampling locations were chosen by the system or if a site was deemed unsuitable due to safety reasons. Each land-use type had 5–6 sites randomly allocated to them and three sites in each of the traffic and dead animal levels, with a minimum 2 km distance between points using the generate random points tool in ArcMap, stored in the system in UTM NAD 83 Zone 17. Where possible, the goal was to have sites evenly distributed in rural, urban, and on the borders of rural and urban areas for each of the land-use elements. Each site was validated using a GPS app (Coordinates—GPS Formatter™, Mapnitude Company Limited, Bangkok, Thailand), physically inspected (validated) to ensure safe access and trap placement, and moved within the immediate local area if the original site was unsuitable, or removed from the sampling design if a local alternative site could not be located. After the validation process, a total of 44 sites were used to sample blow flies, and the livestock farms category was excluded due to the fact that there were too few locations.

### 2.4. Sampling Methods

**Figure 2:**
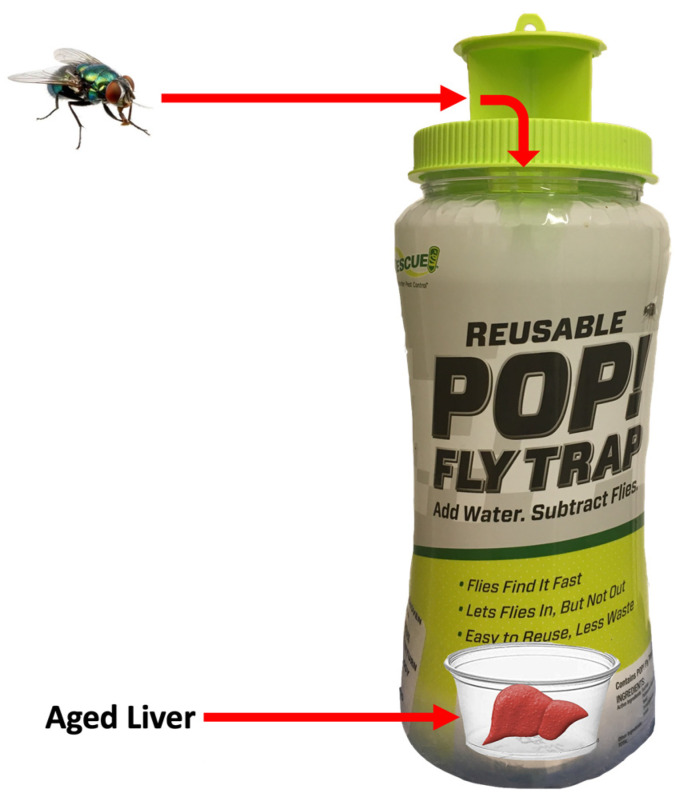
RESCUE POP! Fly Trap, baited with porcine liver.

Adult blow flies were collected using RESCUE POP!**™** Fly Trap (Sterling International, Spokane, WA, USA) during spring (9–18 June 2020), summer (11 August–3 September 2020), and fall (14 October–6 November 2020), with each site containing a trap once for a 24 h period during each season due to the number of sites (44), the driving distance between sites, and the handling time of setting up and collecting traps. Set-up and retrieval occurred between 9:00 and 14:00 h on consecutive days to coincide with the peak of adult blow fly diel activity [27]. Each trap contained a bait of 15 g of porcine liver that had been allowed to decompose for 48 h at a mean temperature of 26 °C (Figure 2). The liver was placed in a 60 mL Solo^®^ cup (Lake Forest, IL, USA) with a layer of distilled water to prevent further drying and to encourage attractiveness inside the traps [28]. Each cup was covered with Better Barriers^™^ Landscape Fabric to prevent the flies from feeding/ovipositing on the liver itself and placed in a RESCUE POP!**™** Fly Trap [29]. After trap retrieval, flies were removed, placed in labelled 50 mm Petri dishes, and stored at −20 °C at the University of Windsor, Department of Integrative Biology until identification. Adult Calliphoridae were identified using Marshall et al. [7] and Jones et al. [30] taxonomic keys.

### 2.5. Geodatabase Data Collection (Buffers) and Creation

Data layers were collected from online geodatabases (available in Table S1, Supplemental Materials). These data layers included: residential, commercial, and wooded areas, waste, various agricultural crop types, traffic volume roads, roadkill density, and streams. The extraction of different data types was performed using ArcMap—Model Builder 10.8x.

#### 2.5.1. Using Buffers for Areal Calculations

Datasets were used to determine the land use allocated to each site’s surrounding area. Using ArcMap, 0.5, 1, and 2 km buffers (circles with a 2 km diameter) were placed around each sampling site. The maximum 2 km buffer size was chosen as 2 km is a known blow fly dispersal distance in a single day [31]. Buffers were used to calculate the area (m^2^) or length of features within the surrounding area. The area within a buffer was calculated for the following layers: residential, commercial, wooded area, waste, and all agriculture crop types. Using the clip tool within ArcMap, the data layers were cut into circles of each buffer size surrounding each sampling site. The total areal unit was calculated for the feature within the clipped circle using calculated geometry. These data were then joined to the site numbers, using the Join to feature tool. Total area units (m^2^) for each site were exported to the overall geodatabase stored in Microsoft Excel^TM^ (Microsoft Corporation, Washington, DA, UDA, 2022).

#### 2.5.2. Using Buffers for Total Length

The length of features within the buffer was calculated for the following data sets: high traffic, low traffic, and medium traffic. The lengths (m) of each feature were extracted within the buffer surrounding each point, and the total road length (m) was calculated for each site using the calculate geometry tool. These total road lengths were added to the overall geodatabase in Microsoft Excel.

#### 2.5.3. Other Data Extracted for Geodatabase

The distance (m) from each site to the nearest stream feature was calculated using a Near Table tool. This tool created a table with the extracted distance variable for each of the sites to the nearest stream feature. Roadkill density values were extracted directly from each sampling site using the Sample tool. The data were added to the overall geodatabase in Microsoft Excel.

#### 2.5.4. Crop Type Combinations for Geodatabase

Original areal crop data included canola, corn, dry bean, soybean, sugar beets, other vegetables, oats, orchards, potatoes, tomatoes, vineyards, and wheat; however, some crop types were only located in a handful of locations (canola, sugar beets, oats, potatoes). Categories were created on the basis of pollen, nectar, likelihood of aphid honeydew, and fruit production as potential protein and sugar sources for blow flies, resulting in (1) wheat/corn/oats (no nectar, wind-pollinated); (2) canola/soybean/dry bean (high pollen, nectar, some aphids); (3) tomatoes/potatoes (pollen, no nectar); (4) orchards/vineyards (high pollen, nectar, and fruit); and (5) sugar beets/other vegetables (aphids).

### 2.6. Statistical Analyses

#### 2.6.1. Diversity Metrics

Mean alpha diversity was calculated to determine the mean number of species in a trap for each land-use type, traffic volume, and roadkill density, with all crop types pooled. Beta diversity was calculated using the species difference between sites within each of the land-use types (with crop types pooled), traffic volumes, and roadkill densities. Gamma diversity was calculated using the total number of species within each land-use type, traffic volume, and roadkill density. The Shannon diversity index [32] was used to calculate the proportion of each blow fly species within each of the land-use types. The Pielou evenness index [33] measured the distribution of blow fly species within each land use type. One-way ANOVAs were performed on the means of the alpha diversity, the Shannon diversity, and the Pielou diversity for each of the 44 sites using IBM SPSS Statistics^™^ for Macintosh, Version 28.0 (IBM Crop, Armonk, NY, USA).

#### 2.6.2. Multiple Regression Model Development

Stepwise regression and adaptive lasso regression were used to assess prospective models correlating the number of blow fly species captured per site or the number of blow fly adults captured per site with multiple geodatabase variables using the Fit Model—Stepwise and Fit Model—General Regression functions with JMP^®^ Pro 18.02. Potential stepwise regression models (*p* < 0.05) were first assessed using corrected Akaike Information Criterion (AICc) and Root Mean Square Error (RMSE) to select candidate models with the lowest combined AICc and RMSE. Variance inflation factor (VIF) was used to detect multicollinearity among geodatabase variables, excluding any models with variables with a VIF above 10. Thus, roadkill density and the three traffic levels were excluded from the analysis in favor of keeping total road length which performed better in side-by-side model comparisons. The independent relative contribution of each variable (not in combination with other variables) to a model was assessed using the Prediction Profiler—Variable Importance function. Candidate models produced by stepwise regression were compared to adaptive lasso regression models using Predictive Modeling—Model Comparison functions within JMP^®^ Pro 18.02 and those with the highest r^2^ and lowest square root of the mean squared prediction error (RASE) were chosen. Principal component analysis of geodatabase variables weighted by species richness or total fly abundance for spring and summer was carried out, and the Scatterplot 3D—PCA function of JMP^®^ Pro 18.02 was used to provide a visual assessment of land-use/landscape patterns and their variation.

## 3. Results

A total of 4639 adult flies among 12 different Calliphoridae species were identified across all traps across all three seasons including *Calliphora livida* Hall, *Calliphora stelviana* (Brauer and Bergestamm)—a new species record for Southern Ontario [34], *Calliphora terraenovae* Macquart, *Calliphora vicina* Robineau-Desvoidy, *Cochliomyia macellaria* (Fabricius), *Cynomya cadaverina* Robineau-Desvoidy, *Lucilia coeruleiviridis* Macquart, *Lucilia illustris* (Meigen), *Lucilia sericata* (Meigen), *Lucilia silvarum* (Meigen), *Phormia regina* (Meigen), and *Protophormia terraenovae* (Robineau-Desvoidy).

Alpha, beta, gamma diversity, Shannon diversity index, and Pielou’s evenness index were calculated for each season. The overall number of species (gamma diversity) across sites, regardless of land-use type was eight species in the spring with an overall trap catch of 1530 from 41 of 44 sites, nine species in the summer with an overall trap catch of 3068 flies from all 44 sites, and six species in the fall, even with the drastically lower trap catch of only 41 flies from only 13 sites. Beta diversity, reflecting the change in species between the land-use types within a season was zero species in spring, two species in summer, and one species in fall. Mean alpha diversity, or the mean species per trap within a land-use type, differed between each season, with the highest number of species in the summer (4.2), followed by spring (3.2), and the lowest in the fall (0.6) (F_2, 129_ = 60, *p* < 0.001). The mean Shannon diversity index reflecting the proportion of each blow fly species within each land-use type was highest in spring (1.60), then in summer (1.41), and lowest in fall (1.14) (F_2, 96_ = 6.2, *p* = 0.003). Pielou’s evenness index of the distribution of those species within a land-use type did not differ between seasons, with 0.77 in the spring, 0.68 in the summer, and 0.64 in the fall (F_2, 76_ = 0.93, *p* =0.4).

Land-use/landscape variables exhibited almost twice the correlation (spring: r^2^ = 0.38 to 0.40; summer: r^2^ = 0.13 to 0.23) with blow fly species richness in the spring than in the summer, despite lower mean alpha diversity (Table 1). Best-fit multivariate models included more variables in the spring (6 to 8) than in the summer (2 to 5). Species richness per site was not described by any multivariate models in the fall due to insufficient sample size compared to land-use/landscape variables. However, when species richness per site was assessed across all three seasons, models consisting of 5 to 7 geodatabase variables demonstrated a low correlation (r^2^ = 0.11 to 0.14) at the two smallest buffer sizes. Species richness models based on 0.5 km buffers provided the highest correlation values, lowest root mean square error, and similar prediction error (AIC_c_) to that of the larger buffer sizes (Table 1).

**Figure 3:**
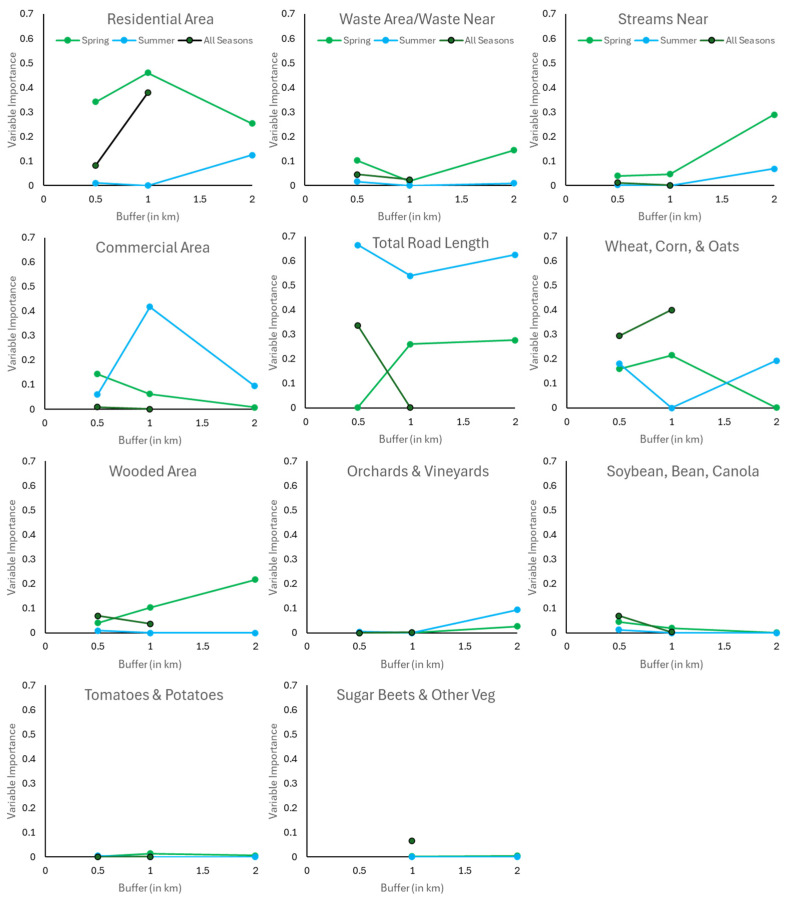
Importance index of individual land-use/landscape variables to blow fly species richness per site multivariate regression models for the spring, summer, and across all three seasons (spring to fall) in Windsor–Essex County, Ontario, CA.

Land-use/landscape variable importance to multivariate models of blow fly species richness varied across buffer size and season, with a frequent inflection point at the 1 km buffer (Figure 3), which was also reflected in the lower r^2^ value of models generated at the 1 km buffer scale (Table 1) compared to 0.5 and 2 km buffers. In the spring, residential areas consistently contributed to species richness models, regardless of buffer size. At 0.5 km buffer scale, commercial area, wheat/corn/oats, nearest distance to waste, and nearest distance to streams provided the next greatest contributions in the spring, whereas at 2 km, total road length, wooded area, nearest distance to streams, and waste area joined residential area to provide the greatest correlation with species richness. In contrast, residential areas contributed far less to species richness models in the summer, whereas total road length was consistently important, followed by commercial areas across all three buffer sizes.

Land-use/landscape variable contribution to species richness models across all three seasons was not additive from spring and summer. Indeed, while the pattern of contribution by individual variables most closely followed that of spring, total road length did not contribute to species richness models at 1 km when all seasons were considered, despite being an important variable in spring and summer at that buffer distance. The wooded area also exhibited the opposite pattern in spring compared to across all seasonsTotal fly abundances exhibited almost twice the correlation (spring: r^2^ = 0.34 to 0.54; summer: r^2^ = 0.22) with land-use/landscape variable in the spring than in the summer (Table 2). Multivariate models included more land-use/landscape variables in the spring (5 to 8) than in the summer (4). However, only the largest buffer of 2 km provided a best-fit multivariate model in the summer. Total fly abundance per site was not described by any multivariate models in the fall due to insufficient sites with a successful fly trap catch; however, when total fly abundance per site was assessed across all three seasons, models consisting of 5 to 8 land-use/landscape variables demonstrated a correlation similar to that of summer (r^2^ = 0.16 to 0.22), despite the greater number of variables included in the all-season models. Total fly abundance models based on 0.5 km buffers provided higher correlation values, lower root mean square error, and similar prediction error (AIC_c_) to that of the larger buffer sizes (Table 2).

**Figure 4:**
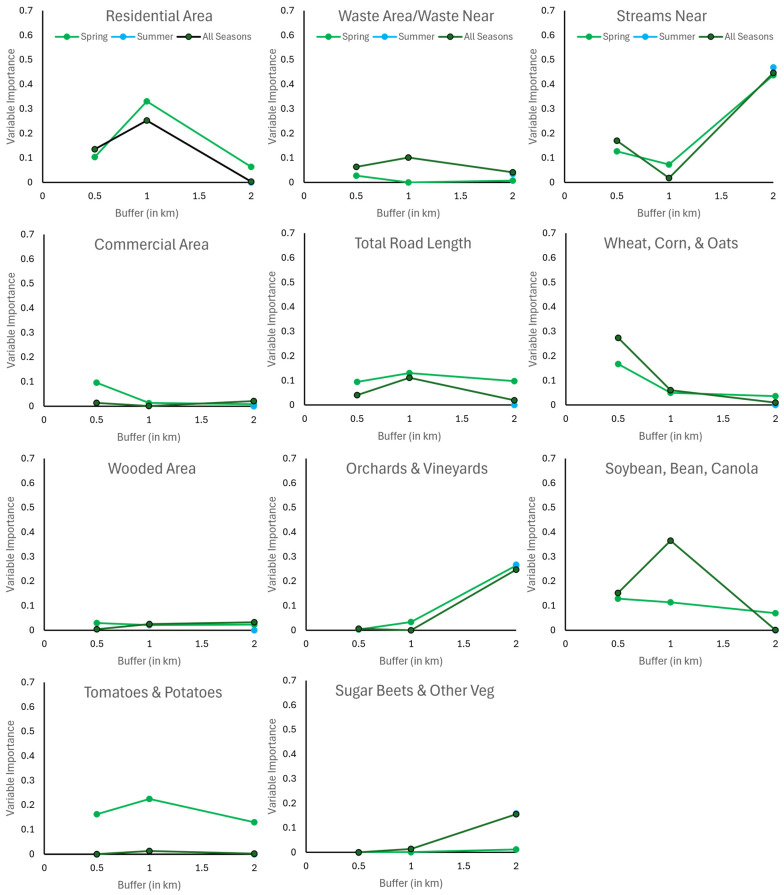
Importance index of individual land-use/landscape variables to blow fly total abundance per site multivariate regression models for the spring, summer, and across all three seasons (spring to fall) in Windsor–Essex County, Ontario, CA.

As with species richness, land-use/landscape variable contribution to multivariate models of blow fly abundance varied across buffer size, with a frequent inflection point at the 1 km buffer (Figure 4). Despite the contribution of summer and fall blow fly abundance trap data into the all-season models, variable contribution followed the same pattern as in spring, with the exception of soybean/dry bean/canola. In the summer, the nearest distance to streams, orchards/vineyards, and sugar beets/other vegetables were important fly abundance model contributors. These same variables were important in the spring at the 2 km buffer distance, together with tomatoes/potatoes, soybean/dry bean/canola, total road length, and residential area.

Within the best-fit multivariate models for species richness and total fly abundance, wooded area, total road length, wheat/corn, and soybean/dry bean/canola were always negatively correlated, whereas distance to streams, tomatoes/potatoes, orchards/vineyards (with one exception), and sugar beet/other vegetables were always positively correlated with species richness or total fly abundance (Table 1 and Table 2). In contrast, residential areas and commercial areas were negatively correlated in the spring but positively correlated in the summer with blow fly species richness and total fly abundance.

The relationship between waste and blow fly species richness or total fly abundance was complicated by both season and buffer distance. It was overall positively correlated with species richness when examined across all seasons (Table 1); however, for fly abundance across all seasons, the waste area was negatively correlated at 2 km and distance to waste was positively correlated at shorter buffer distances (Table 2). Spring species richness was negatively correlated with the waste area at the 2 km buffer distance, but the distance to waste was positively correlated at the 0.5 and 1 km buffer distances. Summer species richness and total abundance were both negatively correlated with waste.

**Figure 5:**
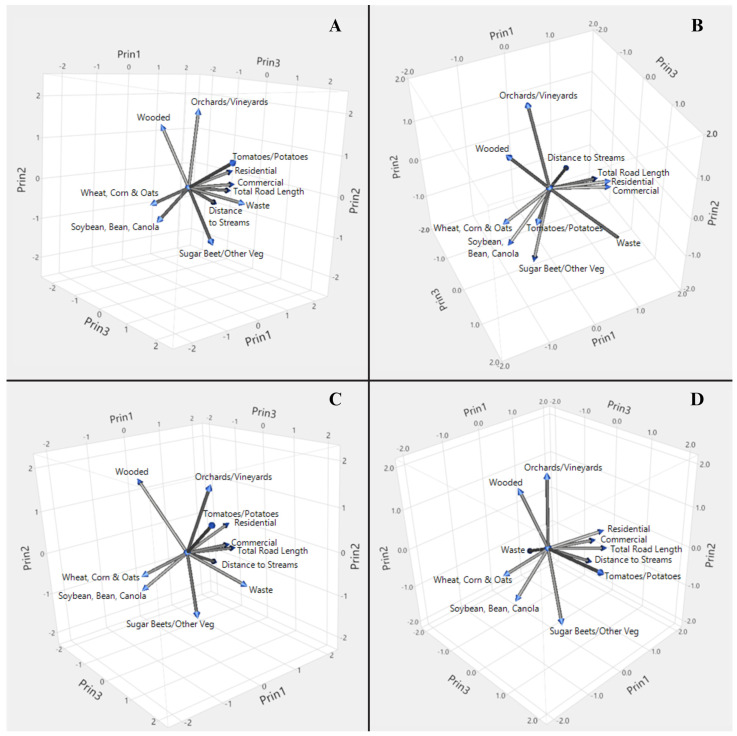
Principal component analysis of land-use/landscape variables weighted by (**A**) blow fly species richness in spring; (**B**) total blow fly abundance in spring; (**C**) blow fly species richness in summer; and (**D**) total blow fly abundance in summer.

Three principal components explain 72% (spring) and 69% (summer) of the overall variance within land-use/landscape variables relating to blow fly species richness and 79% (spring) and 71% (summer) of the overall variance within land-use/landscape variables relating to total fly abundance in spring (Figure 5). For both blow fly species richness and total fly abundance in spring and summer, the first principal component had large (>0.30 eigenvector) positive associations with residential area, commercial area, total road length, distance to streams and large negative associations with wheat/corn/oats and soybean/bean/canola. Whereas the second component had large positive associations with orchards/vineyards and the wooded area, and a large negative association with sugar beets/other vegetables for both species richness and total fly abundance, tomatoes/potatoes were also positively associated (>0.30 eigenvector) but only for blow fly species richness.

However, the third principal component differed between blow fly species richness and total fly abundance in both the spring and summer (Figure 5). In the spring and summer for species richness, and spring for total fly abundance, the third component had a large (>0.30 eigenvector) positive association with waste, but in the summer for total fly abundance, the third component had a large negative association with waste. In the spring and summer for species richness, and summer total fly abundance, the third component had large positive associations with tomatoes/potatoes and sugar beets/other vegetables. The third component of summer species richness also exhibited a large positive association with orchards/vineyards and a large negative association with wooded areas. In contrast, the third component of spring total fly abundance only exhibited a large negative association with distance to streams.

## 4. Discussion

It is not surprising that season influenced blow fly species richness and total fly abundance. The poor trap catch in the fall was due to cool temperatures and the rainy weather conditions. During the 2020 fall trapping period from October 15 to November 6, Windsor–Essex County experienced 6 days with sustained winds above 29 kph (all 24 days with max wind gusts higher than 29 kph), mean highs of 13 °C and lows of 4 °C, and 10 days of rain (Government of Canada Historical Weather Data, Windsor A weather station). Rain [35,36] and winds above 29 kph prevent flight by blow flies [35,37,38]. Yet, flight is possible between wind gusts and periods of rain [39]. Within these limits, some blow fly species are more sensitive to wind and barometric pressure than others [40]. In contrast, the greatest number of species and fly abundance occurred in summer. Unexpectedly, there were no significant multivariate models relating total fly abundance and land-use/landscape features at the two smallest buffer distances. In all other instances, landscape regression models of species richness and abundance developed using the smallest buffer of 0.5 km outperformed 1 km and 2 km buffers with higher correlations and lower root mean square errors, suggesting that 0.5 km would be a reasonable buffer size for future studies. In contrast, Langer et al. [13] found that a 1 km buffer (0.5 km radius) of sample sites was the most effective scale to discern the effects of landscape; however, they limited their analysis to urban versus rural land use.

Further support for the use of the 0.5 km buffer distance was in the response of waste variables within the models. Given the importance of animal protein sources for all carrion-consuming blow fly species as both an oviposition site and food for offspring, as well as nutrition for adults for gonad development, landfill and waste treatment centres, we predicted the presence of this land-use type would be positively correlated with blow fly species richness and abundance. This prediction was upheld for models using smaller buffers in the spring, and likely fall given the all-season result. However, in models using the 2 km buffer scale in the spring or all buffer sizes in the summer, waste was negatively correlated with blow fly species richness and abundance. It is not clear why blow flies would not be positively correlated with waste; however, this seasonal change may be due to a species shift from waste-using species to less-waste-associated species, a shift in use by some of the same species to resources associated with other land-use types, or a combination of both. Given that both residential and commercial land uses are negatively correlated with blow fly species richness and abundance in the spring, but positively correlated in the summer, it is possible some species change from finding resources in waste land-use areas to residential and commercial-based resources such as home garbage and restaurant dumpsters. Typically, landfills and waste treatment areas are located away from areas of human habitation due to odour complaints.

Despite our predicted positive relationship of blow fly species richness and abundance with roads due to the presence of roadkill, this was not the case. Although roadkill does support blow fly nutritional needs for both adults and offspring, it is possible that the positive influence of roadkill at the densities they occurred within Windsor–Essex county was insufficient to overcome the negative impacts of excess localized wind turbulence and insect mortality due to vehicular impact.

Interestingly, wooded areas did not correlate with a higher species richness and abundance but orchards and vineyards did. Wooded areas in Windsor–Essex county are typically Carolinian hardwood forests with few flowering understory plants, whereas orchards and vineyards flower in the spring, often contain clovers or other flowering ground covers between the rows of vines or fruit trees, and fruit that falls and rots, providing sugar and alcohol cues attractive to blow flies. This similar reasoning may explain why tomato fields were always positively correlated with fly species richness and abundance. Tomato plants are transplanted into fields in April–May, with flowering beginning in June and the first harvests in August through to October, with 10,651 acres planted and harvested in Windsor–Essex and Chatham–Kent counties in 2020 [41]; thus, pollen is first, then the fruit is available as protein and sugar sources for blow flies.

In contrast, sugar beets do not produce flowers with pollen or nectar, nor fruit that would provide resources for blow flies, yet blow fly species richness and abundance positively correlate with sugar beet and other vegetables in land-use regression models. We suspect the reason has to do with not the crops themselves, but another insect that is often associated with these vegetables. Sugar beets and other leafy root vegetables such as carrots, onions, or cruciferous vegetables grown in Windsor–Essex county are host to aphids which excrete a sugary waste (honeydew) that numerous other insects use as a sugar source. In addition, blow flies are known pollinators of onions [42], carrots [43], and cruciferous vegetables [44]. Although soybean and dry bean are also host to aphids and we might have expected a positive correlation instead of the negative correlation we obtained, the recent development of aphid-resistant cultivars planted in southern Ontario means reduced aphid populations on the crops [45]. It is unsurprising that the correlation with wheat, corn, and oats was negative given that these crops are wind-pollinated, do not produce nectar, and while they are susceptible to aphids, resistant varieties are available, therefore there may be few resources for blow flies in these fields.

Distance to streams was always positively correlated with blow fly species richness and abundance across landscape regression models, regardless of season. The reason for this may be two-fold. Firstly, closer to streams (which also include wetlands and lakes) should have a higher humidity and blow flies have demonstrated sensitivity to low humidity [46]. Indeed, we have observed faster mortality of adult flies in colony conditions when relative humidity drops (VanLaerhoven, pers. Observations, Windsor, ON, Canada). Secondly, numerous streams in Windsor–Essex are seasonal and dry up, stranding fish. Storms on the lakes and algal blooms often leave dead fish on the shores of Lake St. Clair, Lake Erie, and the Detroit River, as well as in their adjacent wetlands. These processes provide regular sources of fish carrion for blow flies.

Overall, this research has provided predictive multivariate models of land-use and landscape variables correlating with blow fly species richness and total fly abundance, highlighting the positive role of orchards, vineyards, tomatoes, sugar beets and other vegetables together with streams. Either foraging behaviour shifts or changes in species seem to influence the response of blow fly species richness and total fly abundance to residential, commercial and waste land-use types. Buffer sizes of 0.5 km diameter around sampling sites provide the best information for blow fly landscape regression model development. Further research to validate these models and delve into the responses of individual blow fly species will provide insights into resource partitioning between the species across spatiotemporal scales. These models also showcase the importance of detailed studies on adult blow fly behaviour and resource use to better understand how this guild of insects coexists on seemingly rare and ephemeral carrion resources, particularly in the face of increasing habitat fragmentation and anthropogenic impacts on the landscape.

## Figures and Tables

**Table 1:** Blow fly species richness per site best fit multivariate regression models for the spring, summer, and across all three seasons (spring to fall) at three buffer sizes in Windsor–Essex County, Ontario, CA.

Season	Buffer Distance	Number of Predictor Variables	Predictor Variables	r^2^	RASE	AICc	Model Equation
Spring	2 km	6	Residential Area; Wooded Area; Waste Area; Streams Nearest Distance; Tomatoes/Potatoes; Orchards/Vineyards	0.36	1.53	168.31	$\begin{array}{c} =4.45555763702445(-0.00000042023598902*Residential)\\ \;\;\;\;\;\;+(-0.0000010814139366847*Wooded)\\ \;\;\;\;\;\;+(-0.0000086946880007904*Waste)\\ \;\;\;\;\;\;+(0.0843545035492451*StreamsNearDist)\\ \;\;\;\;\;\;+(0.0000011768508513508*Tomatoes/Potatoes)\\ \;\;\;\;\;\;+(0.0000333492106828839*Orchards/Vineyards) \end{array}$
	1 km	6	Residential Area; Wheat/Corn; Wooded Area; Commercial Area; Streams Nearest Distance; Tomatoes	0.38	1.48	169.23	$\begin{array}{c} =6.59690099303542+(-0.000002459112448193*Residential)\\ \;\;\;\;\;\;\;\;+(-0.0000067638388054936*Commercial)\\ \;\;\;\;\;\;\;\;+(-0.0000029933177235215*Wooded)\\ \;\;\;\;\;\;\;\;+(0.00147114381121544*StreamsNearDist)\\ \;\;\;\;\;\;\;\;+(0.0000064564897299816*Tomatoes)\\ \;\;\;\;\;\;\;\;+(-0.0000025533070545677*Wheat/Corn) \end{array}$
	0.5 km	8	Commercial Area; Residential Area; Wooded Area; Streams Nearest Distance; Waste Nearest Distance; Tomatoes; Soybean/Bean; Wheat/Corn	0.40	1.46	174.9	$\begin{array}{c} =5.13241947629962+(-0.0000187646837567535*Commercial)\\ \;\;\;\;\;\;\;\;+(-0.0000064416683241554*Residential)\\ \;\;\;\;\;\;\;\;+(-0.0000078638863008571*Wooded)\\ \;\;\;\;\;\;\;\;+(0.00130467021800771*StreamsNearDist)\\ \;\;\;\;\;\;\;\;+(0.000288921628136109*WasteNearDist)\\ \;\;\;\;\;\;\;\;+(0.0000036939961288755*Tomatoes)\\ \;\;\;\;\;\;\;\;+(-0.0000026083133909814*Soybean/Bean)\\ \;\;\;\;\;\;\;\;+(-0.0000064412481594623*Wheat/Corn) \end{array}$
Summer	2 km	5	Total Road Length; Commercial Area; Streams Nearest Distance, Waste Area; Residential Area	0.20	1.43	169.8	$\begin{array}{c} =4.35798867950955+(0.0000005574733357155*Residential)\\ \;\;\;\;\;\;\;\;+(0.0000025354173862936*Commercial)\\ \;\;\;\;\;\;\;\;+(-0.0000051433316359231*Waste)\\ \;\;\;\;\;\;\;\;+(-0.0000494150373759237*TotalRoadLength)\\ \;\;\;\;\;\;\;\;+(0.0869866235005841*StreamsNearDist) \end{array}$
	1 km	2	Total Road Length; Commercial Area	0.13	1.5	169.83	$\begin{array}{c} =4.49211687923328+(0.0000074001904107543*Commercial)\\ \;\;\;\;\;\;\;\;+(-0.0000577432826377691*TotalRoadLength) \end{array}$
	0.5 km	4	Commercial Area; Waste Nearest Distance; Total Road Length; Wheat/Corn	0.23	1.41	169.6	$\begin{array}{c} =6.2958010766851+(0.0000109917942210878*Commercial)\\ \;\;\;\;\;\;\;\;+(-0.000117060352766665*WasteNearDist)\\ \;\;\;\;\;\;\;\;+(-0.000308315229999502*TotalRoadLength)\\ \;\;\;\;\;\;\;\;+(-0.0000056914244402216*Wheat/Corn) \end{array}$
All Seasons	2 km	.	no model	.	.	.	no model
	1 km	5	Wheat/Corn; Residential Area; Sugar Beets/Other Vegetables; Wooded Area; Waste Nearest Distance	0.11	1.71	398.89	$\begin{array}{c} =4.81550477429406+(-0.0000010241884127129*Residential)\\ \;\;\;\;\;\;\;\;+(-0.0000009019859640548*Wooded)\\ \;\;\;\;\;\;\;\;+(0.0000792159517946115*WasteNearDist)\\ \;\;\;\;\;\;\;\;+(0.000009922011319326*SugarBeets/OtherVeg)\\ \;\;\;\;\;\;\;\;+(-0.0000014705124567248*Wheat/Corn) \end{array}$
	0.5 km	7	Wheat/Corn; Total Road Length; Wooded Area; Soybean/Bean; Residential Area; Waste Nearest Distance; Streams Nearest Distance	0.14	1.69	400.72	$\begin{array}{c} =5.4760657997637+(-0.0000022816368234727*Residential)\\ \;\;\;\;\;\;\;\;+(-0.0000063838467838825*Wooded)\\ \;\;\;\;\;\;\;\;+(0.0004914390627044*StreamsNearDist)\\ \;\;\;\;\;\;\;\;+(0.00013871774671848*WasteNearDist)\\ \;\;\;\;\;\;\;\;+(-0.000185918572049233*TotalRoadLength)\\ \;\;\;\;\;\;\;\;+(-0.0000019422066884166*Soybean/Bean)\\ \;\;\;\;\;\;\;\;+(-0.0000059775812436732*Wheat/Corn) \end{array}$

Note: For rows with no model, no data is indicated by periods.

**Table 2:** Blow fly total abundance per site best-fit land-use/landscape multivariate regression models for the spring, summer, and across all three seasons (spring to fall) at three buffer sizes in Windsor–Essex County, Ontario, CA.

Season	Buffer Distance	Number of Predictor Variables	Predictor Variables	r^2^	RASE	AICc	Model Equation
Spring	2 km	5	Streams Nearest Distance; Orchards/Vineyards; Tomatoes/Potatoes; Residential Area; Wooded Area	0.34	39.5	435.13	$\begin{array}{c} =21.214615497128+(-0.00000546415066459*Residential)\\ \;\;\;\;\;\;\;\;+(-0.000009654044102469*Wooded)\\ \;\;\;\;\;\;\;\;+(2.50946108974846*StreamsNearDist)\\ \;\;\;\;\;\;\;\;+(0.000103367172977714*Tomatoes/Potatoes)\\ \;\;\;\;\;\;\;\;+(0.00221383639449568*Orchards/Vineyards) \end{array}$
	1 km	8	Tomatoes; Residential Area; Streams Nearest Distance; Soybean/Bean; Orchards/Vineyards; Wheat/Corn; Wooded Area; Commercial Area	0.47	35.3	445.81	$\begin{array}{c} =93.4743577411838+(-0.0000550290464246224*Residential)\\ \;\;\;\;\;\;\;\;+(-0.0000956628956588898*Commercial)\\ \;\;\;\;\;\;\;\;+(-0.0000452057668526173*Wooded)\\ \;\;\;\;\;\;\;\;+(0.0462279999375651*StreamsNearDist)\\ \;\;\;\;\;\;\;\;+(0.00053253990798335*Tomatoes)\\ \;\;\;\;\;\;\;\;+(-0.0000269363424359093*Soybean/Beans)\\ \;\;\;\;\;\;\;\;+(0.00212297168742968*Orchards/Vineyards)\\ \;\;\;\;\;\;\;\;+(-0.0000354471906113575*Wheat/Corn) \end{array}$
	0.5 km	8	Tomatoes; Soybean/Bean; Wheat/Corn; Streams Nearest Distance; Commercial Area; Residential Area; Wooded Area; Total Road Length	0.54	32.9	430.19	$\begin{array}{c} =102.924552247006+(-0.000442034247567997*Commercial)\\ \;\;\;\;\;\;\;\;+(-0.000102186867069393*Residential)\\ \;\;\;\;\;\;\;\;+(-0.000168760505244167*Wooded)\\ \;\;\;\;\;\;\;\;+(0.0563926213642998*StreamsNearDist)\\ \;\;\;\;\;\;\;\;+(-0.00428404391298643*TotalRoadLength)\\ \;\;\;\;\;\;\;\;+(0.000829786653903844*Tomatoes)\\ \;\;\;\;\;\;\;\;+(-0.000110221622924982*Soybean/Bean)\\ \;\;\;\;\;\;\;\;+(-0.000184516919286672*Wheat/Corn) \end{array}$
Summer	2 km	4	Streams Nearest Distance; Orchards/Vineyards; Sugar Beets/Other Vegetables; Waste Area	0.22	52.6	477.22	$\begin{array}{c} =41.390646459758+(-0.00012440586639236*Waste)\\ \;\;\;\;\;\;\;\;+(2.76515257710583*StreamsNearDist)\\ \;\;\;\;\;\;\;\;+(0.000340973709786021*SugarBeets/OtherVeg)\\ \;\;\;\;\;\;\;\;+(0.00238559732842547*Orchards/Vineyards) \end{array}$
	1 km	.	no model	.	.	.	no model
	0.5 km	.	no model	.	.	.	no model
All Seasons	2 km	5	Wooded Area; Waste Area; Streams Nearest Distance; Orchards/Vineyards; Sugar Beets/Other Vegetables	0.16	51.5	1044.5	$\begin{array}{c} =30.6508589736018+\left(-0.0000096918476370991*Wooded\right)\\ \;\;\;\;\;\;\;\;+\left(-0.000104672832686726*Waste\right)\\ \;\;\;\;\;\;\;\;+\left(2.12237499557081*StreamsNearDist\right)\\ \;\;\;\;\;\;\;\;+\left(0.00160002341045293*Orchards/Vineyards\right)\\ \;\;\;\;\;\;\;\;+(0.000282587272132582*SugarBeets/OtherVeg \end{array}$
	1 km	8	Residential Area; Soybean/Bean; Wheat/Corn; Waste Nearest Distance; Wooded Area; Streams Nearest Distance; Sugar Beet/Other Vegetables; Tomatoes	0.17	50.8	1070.4	$\begin{array}{c} =88.5051751764908+(-0.000041754835255912*Residential)\\ \;\;\;\;\;\;\;\;+(-0.0000399829592344151*Wooded)\\ \;\;\;\;\;\;\;\;+(0.0256449866765127*StreamsNearDist)\\ \;\;\;\;\;\;\;\;+(0.00707367287204112*WasteNearDist)\\ \;\;\;\;\;\;\;\;+(0.00014687162660354*Tomatoes)\\ \;\;\;\;\;\;\;\;+(-0.0000359970857257758*Soybean/Beans)\\ \;\;\;\;\;\;\;\;+(0.000284530400228854*SugarBeets/OtherVeg)\\ \;\;\;\;\;\;\;\;+(-0.0000334814552843748*Wheat/Corn) \end{array}$
	0.5 km	8	Wheat/Corn; Streams Nearest Distance; Soybean/Bean; Residential Area; Waste Nearest Distance; Commercial Area; Total Road Length; Orchards/Vineyards	0.22	49.4	1065	$\begin{array}{c} =82.601159923182+(-0.00019111975286374*Commercial)\\ \;\;\;\;\;\;\;\;+(-0.0000986377661186682*Residential)\\ \;\;\;\;\;\;\;\;+(0.0533247455524591*StreamsNearDist)\\ \;\;\;\;\;\;\;\;+(0.00556745665979364*WasteNearDist)\\ \;\;\;\;\;\;\;\;+(-0.00263997477694183*TotalRoadLength)\\ \;\;\;\;\;\;\;\;+(-0.0000949264630075459*Soybean/Bean)\\ \;\;\;\;\;\;\;\;+(-0.00019706650279611*Wheat/Corn)\\ \;\;\;\;\;\;\;\;+(-0.00260644439766962*Orchards/Vineyards) \end{array}$

Note: For rows with no model, no data is indicated by periods.

## Data Availability

Dataset available upon request from the authors.

## Supplementary Materials

The following supporting information can be downloaded at: https://www.mdpi.com/article/10.3390/insects15100822/s1. Table S1 provides a list of the data sources and associated website links to create the geodatabase layers used in ArcMap.
