# Supplementary material for: Modelling Blow Fly (Diptera: Calliphoridae) Spatiotemporal Species Richness and Total Abundance Across Land-Use Types
Source: Insects. 2024 Oct 20;15(10):822. doi: 10.3390/insects15100822 (PMC11508989; doi:10.3390/insects15100822)
Supplement: Supplementary file 1 [file insects-15-00822-s001.zip › insects-3237880-supplementary.pdf]

Table S1. Description of data sources and attribute fields relevant to the sampling protocol and relevant data extraction. Websites for those that were sourced online are linked in the Website column.

Parameter	Variable Type	Unit	Attribute Field	Variable Category	Source Layer	Data Source	Websites
**Land-use**	Residential	m	PROP_CODE	301, 302, 303, 304, 305, 306, 307, 309, 311, 313, 314, 322, 332, 333, 334, 335, 336, 340, 341, 350, 352, 360, 361, 366, 368, 370, 371, 372, 373, 374, 375, 378, 379, 380, 383, 385, 386, 601, 602, 605, 608, 610, 611, 621, 623, 624, 625, 626, 627, 630, 631, 632	Land_Parcels_UTM83	City of Windsor, 2019<br><br>County of Essex, 2019	https://opendata.citywindsor.ca/Details/204
	Commercial	m	PROP_CODE	400, 401, 402, 403, 405, 406,	Land_Parcels_UTM83	City of Windsor, 2019	https://opendata.citywindsor.ca/Details/204

				407, 408, 409, 410, 411, 412, 413, 414, 415, 416, 417, 422, 423, 425, 426, 427, 428, 429, 430, 431, 432, 433, 434, 441, 444, 445, 446, 447, 448, 450, 451, 460, 461, 462, 465, 470, 471, 472, 473, 475, 476, 477, 478, 514, 516, 521, 535, 575, 580, 621, 704, 705, 710, 711, 703, 718, 720, 722, 725, 726, 733		County of Essex, 2019	
	Waste	m	N/A	N/A	_ags _DMTI_ 2019_ CMCS _ LiquidDepotDumpRegion ;	Scholars GeoPortal, 2020	**Windsor:** https://geo2.scholarsportal.info/#r /details/_uri@=18787362 **Essex County:** https://geo2.scholarsportal.info/#r

					_ags _DMTI_ 2019_ CMCS _ SolidDepotDumpRegion		/details/_uri@=6232871
	Wooded Area	m	N/A	N/A	WOODAREA	Ontario GeoHub, 2012	https://geohub.lio.gov.on.ca/datasets/lio::wooded-area/explore?location=50.926000%2C-84.745000%2C4.98
	Livestock Farms	# of livestock farms	Livestock Farms	Addresses	N/A	N/A	
**Traffic**	Roads	m	RDCLASS	All classes	Street_Centerline_UTM83	City of Windsor Open Data Catalogue, 2019<br><br>County of Essex open data catalogue, 2019	**Windsor:** https://opendata.citywindsor.ca/details/215<br>**Essex County:** https://opendata.countyofessex.ca/datasets/essexcounty::streetcenterline/about
	Traffic Volume (Low, Medium, High)	m	RDCLASS	**Low:** Local roads<br>**Medium:** Arterial and collector<br>**High:** Highways, and expressways	Street_Centerline_UTM83	City of Windsor Open Data Catalogue, 2019<br><br>County of Essex open data catalogue, 2019	**Windsor:** https://opendata.citywindsor.ca/details/215<br><br>**Essex County:** https://opendata.countyofessex.ca/datasets/essexcounty::streetcenterline/about

**Roadkill**	Dead Animal Removal	Dead animals and their locations	Dead animal density	Low: 1-15<br>Medium: 15-30<br>High: 30-45	N/A	City of Windsor, 2019<br><br>County of Essex, 2019	
